# An examination of the role of Big Five personality traits on employee creativity in Sudanese public universities: a gender-based approach

**DOI:** 10.3389/fpsyg.2025.1556637

**Published:** 2025-05-12

**Authors:** Azadeh Amoozegar, Basma Mohamed Hassan Elsiddig, Mohammad Falahat, Maryam Ikram, Ayman Abdel Rahman Mohamed Ismaeil, Hariharan N. Krishnasamy

**Affiliations:** ^1^Faculty of Education and Liberal Arts, INTI International University, Nilai, Negeri Sembilan, Malaysia; ^2^Limkokwing Graduate School, Limkokwing University of Creative Technology, Cyberjaya, Selangor, Malaysia; ^3^Strategic Research Institute (SRI), Asia Pacific University of Technology and Innovation (APU) Technology Park Malaysia, Kuala Lumpur, Malaysia; ^4^Department of Business Administration, Faculty of Management Sciences, ILMA University, Karachi, Sindh, Pakistan; ^5^Records and Archives Management Department, College of Business Administration, A’Sharqiyah University, Ibra, Oman

**Keywords:** developing countries, innovation, psychological wellbeing, gender inequality, employment policy

## Abstract

**Introduction:**

Sudanese higher education institutions must recognize the influence of Big Five personality traits on employee creativity to foster a workforce that is both innovative and adaptable. These traits play a key role in shaping how employees approach their work and generate new ideas. While studies have explored the link between each of the Big Five personality traits and creativity, the findings have been varied.

**Methods:**

This research employed a cross-sectional correlational approach to examine how the Big Five personality traits influence employee creativity in public universities in Sudan. Participants in the study were randomly selected from five public universities in South Sudan. Data analysis was carried out using SmartPLS 4.

**Results:**

The findings of this research showed that Openness, Agreeableness, and Consciousness significantly influenced creativity, whereas Extraversion and Neuroticism did not. Additionally, the relationship between the Big Five personality traits and creativity was not moderated by gender.

**Discussion:**

Identifying employee personality types and how they influence creativity is crucial for university management when hiring academic staff, especially in developing countries, since it can help them select individuals more likely to excel at research, teaching, and innovation. This knowledge can also inform employment policy to foster an environment conducive to creativity and growth.

## 1 Introduction

In Sudan, higher education places importance on fostering strong connections between academic institutions and the community, while also promoting the spiritual and human values of society to achieve national progress and development (Hibatallah and Rahman, 2020). Higher education play a crucial role in overcoming poverty, as it is essential for driving development and economic growth (Malok, 2012). The philosophy of higher education in Sudan emphasizes the importance of advancing society, meeting its needs, and promoting comprehensive development by equipping individuals with the skills for creativity (Hibatallah and Rahman, 2020). In Sudan, the development of creative abilities is hindered because universities have not succeeded in their essential role of conducting both basic and applied research, thereby failing to meet the country’s needs through their academic endeavors (Hibatallah and Rahman, 2020). Encouraging creativity among academic staffs should be a key focus in higher education institutions, as it is vital for nurturing a culture of knowledge and research within universities (Qahl et al., 2019). Although the advantages of creativity for both personal success and societal progress are well acknowledged, fostering creativity is not emphasized in educational settings (Alencar et al., 2017). Thus, involving academic staff is an appropriate enhancement, as the importance of creativity is not limited to the professional sphere but is also pertinent in academic contexts (Zare and Flinchbaugh, 2019).

Creativity has been defined by Dongell (2021) as the process of generating new and potentially valuable ideas. Scholars and professionals from various fields have increasingly recognized the importance of creativity (Duan et al., 2020; Suifan et al., 2018). The significance of creativity is evident across various fields, including artistic endeavors, educational practices, and commercial enterprises (Kaspi-Baruch, 2017). Further, as a crucial element for competitive advantage, creativity is valued across a variety of tasks, industries, and professions within organizations (Alblooshi, 2018). Creativity is seen as a crucial factor for organizational success and is highly valued by employers during the hiring process (Dongell, 2021). Individuals with a creative disposition exhibit enhanced capabilities in capitalizing on opportunities and addressing challenges more efficiently across their personal and professional domains (Alencar et al., 2017). Due to the critical role creativity plays in both educational settings and professional environments, its antecedents have captivated researchers for a long time (Yao and Li, 2021). Consequently, this topic has garnered extensive focus within scholarly research (Kaspi-Baruch, 2017).

Over the past few decades, there has been a growing consensus among personality psychologists about the framework and understanding of personality. Most researchers agree that five key factors provide a comprehensive classification system for personality traits (Christensen et al., 2014). The Big Five personality traits framework, which includes conscientiousness, extraversion, agreeableness, openness, and neuroticism, was introduced by Goldberg (1992) and has been extensively used in previous research to enhance the understanding of personality structure (Manteli and Galanakis, 2022). Measures of personality aim to capture an individual’s usual ways of thinking, feeling, and acting, as well as the underlying psychological processes—whether visible or hidden—that shape these patterns, which are the most effective and common indicators of creativity (Hornberg, 2022). Kim Nam and Thi Hang Nga (2024) emphasized the importance of individual factors in enhancing creativity, highlighting the significant role of personality traits in this context. Yao and Li (2021), noted that the Big Five personality is frequently applied to examine the link between creativity and personality. Although research has investigated the association between each of the Big Five personality traits and creativity, the results have been inconsistent (Kaspi-Baruch, 2017). Consequently, the ongoing exploration of the relationship between Big Five personality traits and employee creativity continues to yield varied outcomes, leading researchers to advocate for further investigation into this area (Jirásek and Sudzina, 2020; Puryear et al., 2019).

Sudan is rich in different races and cultures and a mixture of Arab and African tribes (Farah Bakhiet and Mohamed, 2022). Sudan’s indigenous sociocultural framework is a complex blend of interactions and integrations among various elements, primarily African, Arab, and Islamic in nature. The people adhere to various religions, speak multiple languages, and lead a wide array of lifestyles (Khaleefa et al., 1996). These aspects play a crucial role in shaping individual behavior, especially in terms of creativity. For example, in Arab cultures, there is a constant pattern of sex differences favoring males over females in creativity scores. Similarly, in Nigeria, males were found to be more flexible than females (Khaleefa et al., 1996). Although there may be differences between males and females in the factors that affect their creativity, research has not reached a definitive agreement on gender-related disparities (Baer and Kaufman, 2008). Most research has shown no gender-based differences in creativity, and those that have identified differences have not observed any consistent patterns (Afu, 2020; Kaufman, 2006; Zare and Flinchbaugh, 2019). However, Al-Srour and Al-Oweidi (2013) reported that women tend to be more creative than men, possibly due to their quieter nature and greater dedication to tasks, which are crucial for fostering creativity. Karwowski et al. (2013) found women succeed less often that men in mature and outstanding creative achievements. While there is no consensus on how creativity differs by gender, this research aims to fill this gap by exploring the creative differences between men and women in a Sudanese setting.

Researchers advocate for continued exploration of the connection between personality and creativity, despite the inconclusive findings and unresolved questions surrounding the topic (Stritmatter, 2023). Identifying the significance of this relationship and developing effective methods for measuring and predicting its practical implications and application are crucial (Kaufman et al., 2022; Puryear et al., 2019). The contradiction in findings calls for more studies across different contexts to offer a supplementary detailed understanding of these relationships (Zare and Flinchbaugh, 2019). Yao and Li (2021) highlight the significance of delving deeper into the connection between personality traits and employee creativity in organizational settings. Although the relationship between personality traits and creativity has been studied, it has not been comprehensively explored in Sudan. Therefore, from an academic perspective, it is vital to simultaneously examine the relationship between the Big Five personality traits and employee creativity, while also analyzing gender differences within the same research framework.

**Figure 1:**
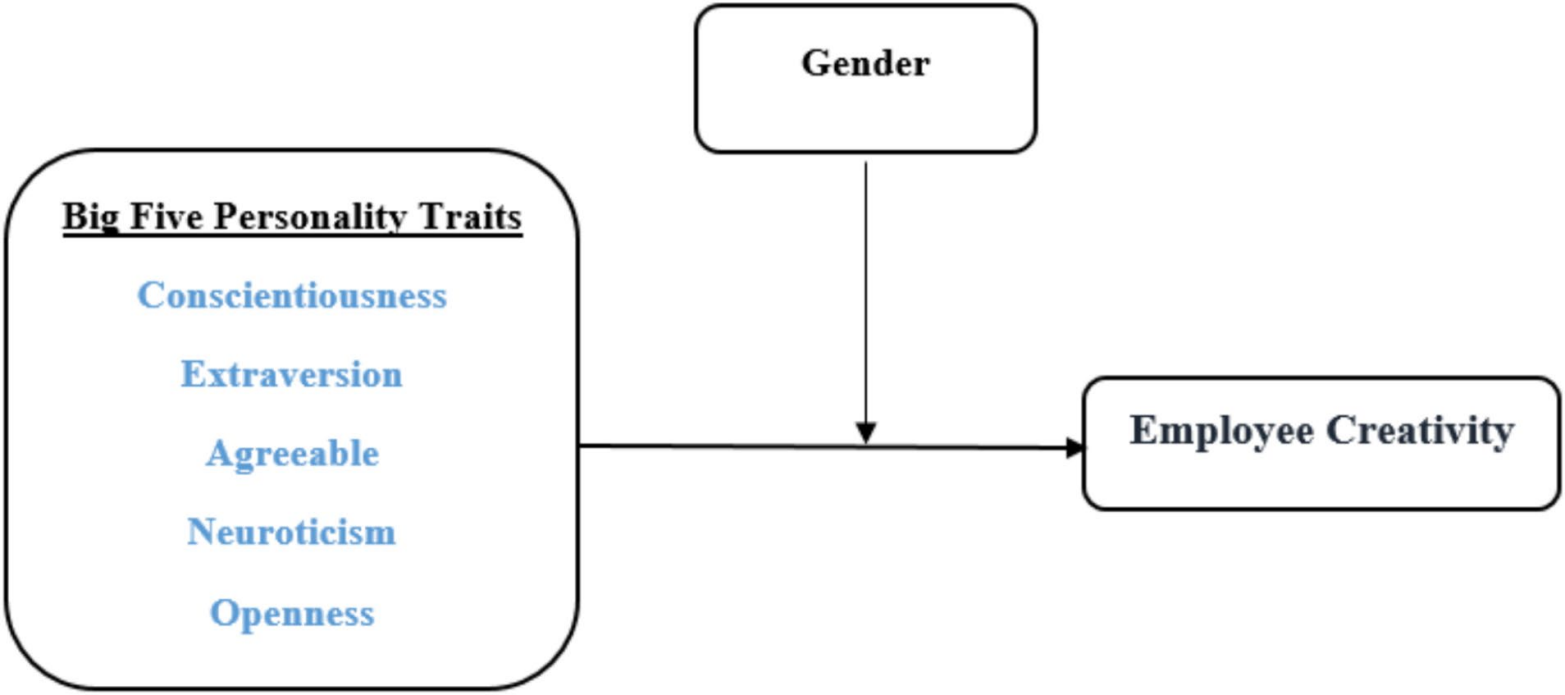
Model of employee creativity.

Upon reviewing the literature studies, it becomes clear that there is a focus on the links between personality traits and employee creativity, often overlooking the role of moderation. Notably, the use of gender as a moderating factor in research models to evaluate inter-relationships and effects is missing. The ways in which gender influence the relationship between personality and creativity is less well understood. Furthermore, many studies concentrate solely on openness to experience, neglecting other traits. Additionally, there is a scarcity of research assessing these effects within the context of developing countries, such as Sudan. Empirical studies investigating these causal relationships among academic staff are also lacking. Jirásek and Sudzina (2020) suggest that it is valuable to replicate earlier studies in different contexts to collectively build a comprehensive set of findings on a specific subject. Therefore, this study seeks to address these research gaps by exploring the connections between the big five personality traits and employee creativity, with gender acting as a moderator (Figure 1). The following sections will delve into the theoretical relationships between personality traits and employee creativity.

## 2 Literature review and hypotheses development

Various approaches exist to explain creativity, especially at the individual level. Employees demonstrate creativity by coming up with new and potentially valuable ideas related to new products, services, production techniques, and administrative procedures that enhance organizational innovation and efficiency (Yao and Li, 2021). Scholars believe that an employee’s creativity is significantly shaped by their personality traits (Kim Nam and Thi Hang Nga, 2024; Zare and Flinchbaugh, 2019). Personality traits are characterized as the aspects of individual differences that lead to consistent patterns in thinking, feeling, and behaving (Stritmatter, 2023). Personality traits refer to psychological characteristics that provide reasons behind behaviors (Abou-Shouk et al., 2022). These characteristics consist of complex set of traits which were grouped into five personality traits. The Big Five personality traits, which include openness to experience, conscientiousness, extraversion, agreeableness, and neuroticism, are widely recognized as the most comprehensive framework for identifying individual differences (Abdullah et al., 2016). While personality traits have received increasing attention in literature, but the extent to which they may influence creativity remains largely unexplored (Amin et al., 2020; Mutlu, 2017), especially among academic staffs. Therefore, scholars recommended that studies on personality and creativity should be pursued further to enhance our comprehension of the impact of personality traits on creative behavior (Stritmatter, 2023; Yao and Li, 2021).

This research responds to Amabile (2017), who proposed that future studies should explore the intricate role personality traits might play in generating creative ideas. Scholars have noted that personality traits have a notable impact on creativity, though the extent and consequences of this influence can differ (Collins and Cooke, 2013; Dongell, 2021; Hornberg, 2022; Zare and Flinchbaugh, 2019). According to trait activation theory, the effect of personality on behavior can change based on the context, resulting in varying connections between personality traits and employee creativity (Manteli and Galanakis, 2022). A key question being explored is whether specific personality traits are demonstrating greater creativity (Jirásek and Sudzina, 2020). Extensive research on creativity has indicated that openness to experience, one of the Big Five personality traits, is the only factor consistently linked to creativity (Samašonok and Juškevičienė, 2022; Stevenson et al., 2021; Kruyen and van Genugten, 2017). However, researchers have pointed out that studies on the other Big Five personality traits (i.e., conscientiousness, extraversion, agreeableness, and neuroticism) have either been overlooked or yielded inconclusive results (Kruyen and van Genugten, 2017; Samašonok and Juškevičienė, 2022). Hence, this research delved into the specifics of individual personality traits and examined their unique components. These elements enhance our current comprehension of the relationship between personality and creativity (Figure 1).

Hong et al. (2020) reported that individuals with high creativity tend to have the following personality traits: extraversion, conscientiousness, and openness. Collins and Cooke (2013) argued in their study that conscientiousness played the most important role in creativity among the Big Five factors. Furnham et al. (2013) reported that only extraversion and openness were positively correlated with creativity. Similarly, the research by Sung and Choi (2009) revealed that extraversion and openness to experience positively affect creative performance, while the influence of other traits is less reliable. A study conducted by Tsai et al. (2024) in the hospitality and tourism industry, found that extraversion and conscientiousness influencing creativity among students. Research has consistently supported the link between openness to experience and creativity among the five personality traits, as evidenced by existing literature and previous studies (Christensen et al., 2014; Karwowski et al., 2013). In contrast, studies focusing on the other Big Five traits—conscientiousness, extraversion, agreeableness, and neuroticism—have either been overlooked or have yielded inconsistent findings (Samašonok and Juškevičienė, 2022; Kruyen and van Genugten, 2017). Consequently, Stritmatter (2023) suggested a re-evaluation and critical analysis of past research to investigate how each of these personality traits might influence the development of creative ideas.

Openness to experience is one of the Big Five personality traits, along with intellectual curiosity, imagination, originality and liberal attitudes (Yao and Li, 2021). Individuals who exhibit a strong tendency for openness to experience are generally more expressive with their emotions and feelings, and are often characterized by their imagination and adventurousness (Barańczuk, 2019). It denotes personality traits such as curiosity, novelty, cultivated, esthetic, sensitivity, independent minded, intellectualism, and creativity (Abdullah et al., 2016). Openness to experience captures an individual’s openness to new ideas, their intellectual or vocabulary skills, and their curiosity, particularly in new and unfamiliar situations (Puryear et al., 2019). Studies show that individuals with higher openness to experience tend to engage more in creative pursuits and exhibit creative behaviors (Silvia et al., 2014). Hornberg (2022), reported that research on creativity has consistently pointed to openness to experience as the only personality trait reliably linked to creativity. Similarly, Samašonok and Juškevičienė (2022), stated that most research studies in this field demonstrate that openness exerts the strongest influence on an individual’s creative expression. Munir and Beh (2016), however, found inconsistent significance levels for the connection between openness to experience and creativity.

Extraversion reflects the degree to which individuals are assertive, dominant, energetic, active, talkative, and enthusiastic (Jirásek and Sudzina, 2020). The dimension of extraversion is characterized by affection or friendliness, a preference for the company of other people, assertiveness, engagement in numerous activities, a desire for stimulation or excitement, and an optimistic or cheerful demeanor (Hornberg, 2022). Highly extraverted individuals tend to be proactive, socially confident, and seek opportunities for positive social interactions (Zare and Flinchbaugh, 2019). According to Sung and Choi (2009), creativity often stems from proactive behavior. Studies suggest that extraversion can stimulate employee curiosity and excitement for seeking new experiences, ultimately boosting creative thinking and performance (Dongell, 2021). Additionally, extraversion may facilitate the exchange of information between coworkers, contributing to the generation of creativity (Chiang et al., 2017). Extroversion is considered a positive indicator of creative abilities (Karwowski et al., 2013; Yesil and Sozbilir, 2013; Zare and Flinchbaugh, 2019). This positive relationship is strongly supported by the consistent outcomes of several prior studies.

Conscientiousness is another personality trait that has produced contradictory findings regarding its relationship with creativity. Conscientiousness is associated with an individual’s perception of self-efficacy, preference for orderliness, individual reliability, desire to achieve, self-discipline, and preference to think carefully before acting (Hornberg, 2022). A person’s conscientiousness is a reflection of their discipline and direction, their goal-setting abilities, and their reliability as individuals (Puryear et al., 2019). As part of being conscientious, one must be persistent, diligent, self-controlled, organized, and have a goal in mind (Stritmatter, 2023; Zare and Flinchbaugh, 2019). High conscientiousness is typically associated with individuals who are organized, competent, achievement-focused, dependable, and disciplined (Barańczuk, 2019; McCrae and Costa Jr, 1989). Such individuals are usually focused on completing their tasks and are less likely to pause the process to consider alternative methods or ideas (Munir and Beh, 2016). According to a study by Kim Nam and Thi Hang Nga (2024) the relationship between personality traits along with employee creativity in Vietnam private companies, conscientiousness was having a direct positive impact on creativity. According to Rothmann and Coetzer (2003), Karwowski et al. (2013), and Silvia et al. (2014), conscientiousness positively affects individual creativity, while Abdullah et al. (2016) reported that highly conscientious individuals are generally less creative.

Agreeableness describes an individual’s trust in other people, the tendency to respond frankly or sincerely, consideration of other people, willingness to forgive, and preferences toward modesty, and sympathy (Hornberg, 2022). Those who score high in agreeableness are typically kind, friendly, and affectionate. They work well with others and emphasize empathy when interacting (Samašonok and Juškevičienė, 2022). In Stritmatter’s (2023) view, agreeableness relates to the more benevolent aspects of human nature, including altruism, care, and emotional support, while the less agreeable traits at the other end include hostility, indifference, selfishness, spite, and jealousy. Individuals with high agreeableness tend to think more collectively and are flexible, sympathetic, and forgiving (Puryear et al., 2019). A high degree of contradiction exists between creativity and agreeableness (Abdullah et al., 2016; Jirásek and Sudzina, 2020). Although few scientific studies have explored the link between agreeableness and individual creativity, it can be assumed that this trait may serve as an indicator of an individual’s creative potential (Kaufman and Beghetto, 2013; Samašonok and Juškevičienė, 2022). However, a study by Karwowski et al. (2013) examining the correlation between the Big Five personality traits, creative personal identity (CPI) and creative self-efficacy (CSE) revealed a negative association among agreeableness and creativity. Similarly, Nguyen et al. (2024) found insignificant relationship between agreeableness and creativity. Individuals with high agreeableness may find it difficult to express novel and inventive ideas, as this could lead to disagreements or challenge established norms (Zare and Flinchbaugh, 2019; Sung and Choi, 2009).

Neuroticism measures an individual’s tendency to be anxious, experience anger and depression, become embarrassed, control urges and desires, and to cope with stressful situations (Hornberg, 2022). Individuals with high neuroticism are prone to negative emotions and distress, which affects their evaluation of experiences. Associated feelings include fear, anxiety, anger, frustration, depression, loneliness, low self-esteem, poor impulse control, and self-consciousness (Stritmatter, 2023). Neurotic individuals are more inclined to mood swings (Kavirayani, 2018) and often experience emotions like anger, stress, nervousness (Irmscher, 2019), as well as anxiety, frustration, and jealousy (Costa Jr and McCrae, 1992). Due to limited emotional regulation abilities, they tend to be anxious, experience distress, avoid situations, and speak less frequently (Zare and Flinchbaugh, 2019). Among the traits studied, neuroticism has been the least connected to creativity in prior research (Jirásek and Sudzina, 2020). Kim Nam and Thi Hang Nga (2024) and Karwowski et al. (2013) found that this personality trait showed an insignificant relationship to individual creativity. Based on the evidence presented above, this study suggests the following hypothesis:

H1: Big Five personality traits (Openness, Neuroticism, Extraversion, Conscientiousness, Agreeableness) have a significant impact on employee creativity.

H1a: Openness has a significant impact on employee creativity.

H1b: Neuroticism has a significant impact on employee creativity.

H1c: Extraversion has a significant impact on employee creativity.

H1d: Conscientiousness has a significant impact on employee creativity.

H1e: Agreeableness has a significant impact on employee creativity.

### 2.1 Moderator variable: gender

Gender differences need to be considered when evaluating personality traits and creativity (Pérez-Luño et al., 2023). Gender differences have not been a major emphasis in creativity or psychological study, despite several studies on the topic (Baer and Kaufman, 2008). Even though clear differences in creative potential are not apparent, women generally achieve fewer mature and exceptional creative accomplishments compared to men (Abra and Valentine-French, 1991). This gap might be attributed to a lack of self-belief in their creative potential among women (Karwowski et al., 2013). While some research suggests that creativity is relatively equal among men and women (Baer and Kaufman, 2008), findings have varied. Consequently, investigating gender as a potential moderator can help clarify this discrepancy. For example, Amabile et al. (2005) reported a significant link between gender and creativity, whereas Liao et al. (2010) found no such relationship. Likewise, Zare and Flinchbaugh (2019) suggested that there were no gender differences in the association involving personality traits and both voice and creativity.

Study by Karwowski et al. (2013) explored gender’s role as a moderator in the association between the Big Five personality traits and creative personal identity (CPI) as well as creative self-efficacy (CSE). The findings of the study indicated the contrasts amongst the predictors of CSE and CPI. Both men and women exhibited a predictive relationship between Openness to Experience, Neuroticism and Conscientiousness and creative self-efficacy. In women, Extraversion was positively linked to creative self-efficacy, whereas Agreeableness showed a negative relationship. Agreeableness negatively impacted creative personal identity solely in women, while Conscientiousness had a positive effect only in men. In both genders, Extraversion, Neuroticism, and Openness were found to predict creative personal identity. Considering the points discussed above, the following hypothesis is proposed by this study:

H2: Gender significantly moderate the relationship between big five personality traits and employee creativity.

## 3 Methods

### 3.1 Research design

The research sample comprises full-time faculty members employed at public universities in South Sudan. In this developing country, there is a lack of studies on employee creativity within higher education settings. In Sudan, higher education emphasizes building strong ties between academic institutions and the community, aiming to uphold the spiritual and human values of society. This collaboration is deemed essential for advancing national development and progress. Given that educational institutions and their academic staff play a crucial role in shaping society, a study on creativity among Sudanese academic members is anticipated to offer valuable insights for other developing nations.

The study employed a quantitative correlational research approach to examine the elements of creativity, the unique aspects of personality traits, their interrelationships, and how creativity is manifested based on these traits. A correlational research design was chosen to determine if and to what extent personality traits were related to creativity among faculty members in South Sudan. According to Stritmatter (2023) a correlational study aims to assess the strength of the relationship between two or more variables and the extent of any statistical connection between them. In this type of study, as noted by Pressman (2016), no variables are manipulated, and the focus is on identifying correlation rather than causation. This study used survey which is one type of correlational studies.

Online survey and convenience sampling has been employed in this study. An online survey was utilized in this study to objectively and scientifically evaluate the Big Five personality traits and creativity levels. Online surveys offer benefits such as ease of use and a sense of privacy compared to other survey methods (Silver et al., 2017). With assistance from the dean and faculty administration, an email invitation was sent out to join the research, and the questionnaire was conducted through Google Forms. A cover letter accompanied the questionnaires, outlining the study’s purpose, ensuring anonymity, and emphasizing confidentiality. Due to confidentiality concerns, the article does not mention the names of higher education institutions. Data collection took place between 1 June 2023 and 31 August 2023. Out of 389 distributed questionnaires, 263 were returned, yielding a response rate of 67.6%. Of the respondents, 65.3% were male, while 34.7% were female.

Data analysis was conducted using SPSS and SmartPLS 4 software. The analytical procedures involved assessing the reliability of the scales with Cronbach’s alpha, evaluating both convergent and discriminant validity, and testing the hypotheses using structural equation modeling (SEM). Furthermore, procedures for evaluating both the measurement model and the structural model were also carried out (Osman et al., 2024). To determine if gender influences the link between personality traits and employee creativity, researchers employed a multi-group analysis and interaction effect analysis using SmartPLS 4 to investigate gender-based differences. The study assessed path coefficients for each gender group and examined the significance of the interaction term.

### 3.2 Measurement scales

The research employed questions from existing scales, which were subsequently modified to fit the study’s particular context. The questionnaire was divided into two sections: Part 1 included demographic questions, while Part 2 concentrated on Big Five personality traits and employee creativity. A pilot test was conducted with 10 lecturers, and the results indicated that no modifications to the questionnaire were necessary.

The scales utilized in this study were adapted from Tussey (2023) and Seo (2021). The Big Five scale, which measures five personality traits (Openness to Experience, Conscientiousness, Extraversion, Agreeableness, and Neuroticism), consists of 44 items derived and modified from Tussey (2023). Several items from the personality traits scale are presented below: “I see myself as someone who is talkative” and “I see myself as someone who is original, comes up with new ideas.” Respondents were instructed to indicate the extent of their agreement or disagreement with each of the 44 items using a five-point Likert scale, ranging from 1 (strongly disagree) to 5 (strongly agree). The Cronbach’s alpha values exceeded 0.9 for five personality types, demonstrating that the instrument possesses outstanding internal consistency. This outcome indicates that the data gathered from the big five personality traits instruments is highly dependable and suitable for further statistical analysis.

The creativity scale, consisting of 13 items adapted from Seo (2021), has a Cronbach’s alpha value of 0.972. Below are some sample items: “academic staffs suggest new ways to achieve goals or objectives” and “academic staff comes up with new and practical ideas to improve performance.” The questions were measured using a 5-point Likert scale, ranging from 1 (strongly disagree) to 5 (strongly agree). Utilizing the Likert scale, which is the most commonly employed self-administered measurement tool, each item was given numerical values to generate quantifiable data, allowing for the assessment of whether statistical significance is present and to what degree.

## 4 Results

### 4.1 Validating lower order Big Five personality traits construct

**Figure 2:**
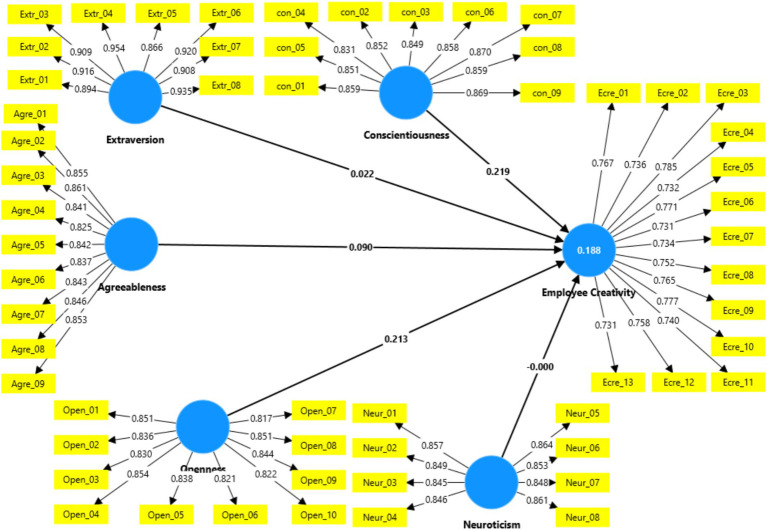
Measurement model.

To evaluate the quality of constructs in this investigation, the measurement model is measured, beginning with the analysis of factor loadings and proceeding to the assessment of construct validity and reliability (Figure 2).

#### 4.1.1 Factor loadings

Factor loading reflects the degree of association between each item in the correlation matrix and a specific principal component. Values range from −1.0 to +1.0, with higher absolute values indicating a more significant correlation between the item and the underlying factor (Pett et al., 2003). In this study, all items had factor loadings above the recommended threshold of 0.50 (Hair et al., 2016), meaning no items were excluded. A summary of the factor loadings is provided in Table 1.

**Table 1:** Factor loading.

	Agreeableness	Conscientiousness	Employee creativity	Extraversion	Neuroticism	Openness
Agre_01	0.855					
Agre_02	0.861					
Agre_03	0.841					
Agre_04	0.825					
Agre_05	0.842					
Agre_06	0.837					
Agre_07	0.843					
Agre_08	0.846					
Agre_09	0.853					
Ecre_01			0.767			
Ecre_02			0.736			
Ecre_03			0.785			
Ecre_04			0.732			
Ecre_05			0.771			
Ecre_06			0.731			
Ecre_07			0.734			
Ecre_08			0.752			
Ecre_09			0.765			
Ecre_10			0.777			
Ecre_11			0.740			
Ecre_12			0.758			
Ecre_13			0.731			
Extr_01				0.894		
Extr_02				0.916		
Extr_03				0.909		
Extr_04				0.954		
Extr_05				0.866		
Extr_06				0.920		
Extr_07				0.908		
Extr_08				0.935		
Neur_01					0.857	
Neur_02					0.849	
Neur_03					0.845	
Neur_04					0.846	
Neur_05					0.864	
Neur_06					0.853	
Neur_07					0.848	
Neur_08					0.861	
Open_01						0.851
Open_02						0.836
Open_03						0.830
Open_04						0.854
Open_05						0.838
Open_06						0.821
Open_07						0.817
Open_08						0.851
Open_09						0.844
Open_10						0.822
Con_01		0.859				
Con_02		0.852				
Con_03		0.849				
Con_04		0.831				
Con_05		0.851				
Con_06		0.858				
Con_07		0.870				
Con_08		0.859				
Con_09		0.869				

##### 4.1.1.1 Reliability analysis

According to Mark (1996) reliability refers to the degree to which a measuring instrument consistently produces stable results. The core of reliability lies in its repeatability—whether the instrument yields the same outcomes when applied repeatedly. Cronbach’s Alpha and Composite Reliability (CR) are the two most frequently used methods for evaluating reliability. The results for both measures are shown in Table 2. Cronbach’s Alpha values ranged from 0.936 to 0.976, and the CR values surpassed the recommended threshold of 0.70 (Hair, 2011), confirming the reliability of the constructs.

**Table 2:** Factor loadings, reliability and AVE for HOC.

	Outer loadings	Alpha	CR	AVE
Extr ← BFPT	0.810	0.754	0.846	0.555
Neur ← BFPT	0.866			
Open_ ← BFPT	0.782			
Con ← BFPT	0.853			
Agre ← BFPT	0.889			

##### 4.1.1.2 Construct validity

Convergent validity measures how well multiple indicators of the same concept align with each other. It implies that valid measures of a concept should show high covariance (Bagozzi et al., 1991). This form of validity is supported when the Average Variance Extracted (AVE) value meets or exceeds 0.50, indicating that the items sufficiently converge to represent the construct (Fornell and Larcker, 1981). In this study, all constructs surpassed the 0.50 AVE threshold, confirming that convergent validity is not an issue. Table 3 presents the AVE values for each construct.

**Table 3:** Construct validity and reliability.

	Cronbach’s alpha	Composite reliability (rho_a)	Composite reliability (rho_c)	Average variance extracted (AVE)
Agreeableness	0.950	0.952	0.957	0.714
Conscientiousness	0.954	0.956	0.961	0.732
Employee creativity	0.936	0.938	0.944	0.566
Extraversion	0.976	1.001	0.976	0.834
Neuroticism	0.946	0.948	0.955	0.727
Openness	0.952	0.953	0.959	0.700

##### 4.1.1.3 Discriminant validity

Discriminant validity assesses how effectively measures of different concepts are differentiated from one another. The underlying principle is that if concepts are truly separate, valid measures of these concepts should show low correlations (Bagozzi et al., 1991).

##### 4.1.1.4 Fornell and Larcker criterion

Following the criterion outlined by Fornell and Larcker (1981), discriminant validity is affirmed when the square root of a construct’s AVE is greater than its correlations with all other constructs. In this study, the square root of the AVE (shown in bold and italics) for each construct exceeded its correlations with other constructs, providing solid evidence for the establishment of discriminant validity.

### 4.2 Validating higher order Big Five personality traits construct (reflective–reflective)

Big Five personality traits were the higher order construct in the study based on five lower order constructs (openness to experience, conscientiousness, extraversion, agreeableness, and neuroticism). The Big Five personality traits are modeled as a Reflective-Reflective higher-order construct in the study because each individual trait (Extraversion, Agreeableness, Conscientiousness, Neuroticism, and Openness) is itself a reflective construct. This means that each trait is represented by its own set of reflective indicators that capture its underlying essence. Consequently, when these five traits are combined into a higher-order model, the overall structure remains reflective-reflective, as the higher-order construct reflects the shared variance of its reflective components.

The validity of higher-order construct was established by evaluating its factor loadings, reliability, and validity. All the indicators for the Big Five personality traits demonstrated factor loadings greater than the minimum acceptable threshold of 0.50 (Nguyen et al., 2024). All items were retained as none had satisfactory factor loadings. The reliability of the higher-order construct was evaluated using Cronbach’s alpha and composite reliability, with both measures surpassing the 0.700 threshold recommended by Wasko and Faraj (2005), thus affirming good reliability (Henseler et al., 2016). The higher-order construct was deemed to have acceptable convergent validity, as its AVE exceeded the 0.500 threshold. The Heterotrait-Monotrait Ratio (HTMT) and the square root of the AVE (Fornell and Larcker, 1981) and Heterotrait-Monotrait Ratio were used to compare the correlations between latent variables in order to evaluate discriminant validity. Given that the square root of the AVE for the construct is greater than its correlations with the other constructs, and the HTMT results (Table 4) indicate that the HTMT ratio remains below the 0.90 threshold, discriminant validity is established for the higher-order construct of the Big Five personality traits.

**Table 4:** Discriminant validity.

HTMT	
	E_creativity
BFPT	0.509

Fornell-Larcker criterion		
	E_creativity	BFPT
E_creativity	0.709	
BFPT	0.421	0.745

### 4.3 Structural model

**Figure 3:**
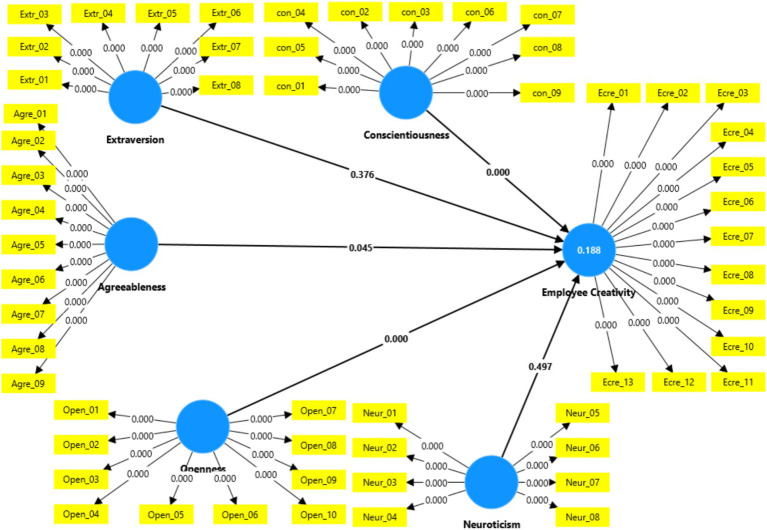
Structural model-LOC.

**Figure 4:**
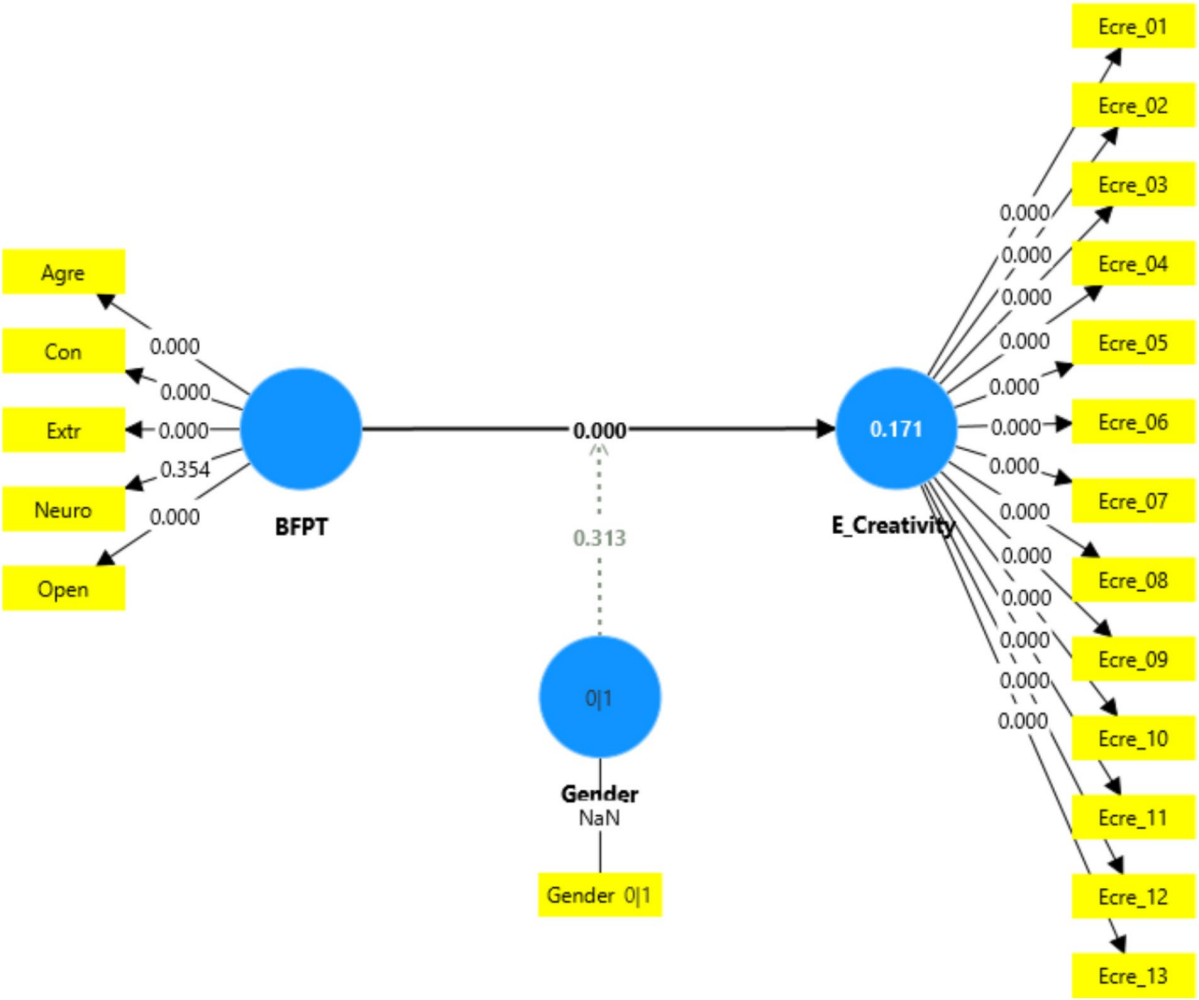
Structural model-HOC.

The findings showed that Openness has significant impact on creativity (*p* < 0.001), thus supporting H1a. According to H1d, positive relationship is predicted between Conscientiousness and employee creativity. The findings demonstrate a significant effect of Conscientiousness on employee creativity (*p* < 0.001), thereby supporting H1d. The results also indicated that Agreeableness has a positive and significant effect on employee creativity, thus, H1e also supported. However, the effect sizes for Neuroticism and Extraversion were not significant (*p* = 0.497 and *p* = 0.376, respectively), indicating that neither is a reliable predictor of creativity. Therefore, H1b and H1c were not supported in this investigation. Table 5 and Figure 3 highlights the path coefficient results illustrating the influence of the Big Five personality traits on employee creativity (Figure 4 and Tables 6, 7).

**Table 5:** Direct effect-LOC.

	*β*	STDEV	*t* statistics	*p* values
Agreeableness → Employee Creativity	0.090	0.052	1.706	0.045
Conscientiousness → Employee Creativity	0.219	0.058	3.791	0.000
Extraversion → Employee Creativity	0.022	0.070	0.318	0.376
Neuroticism → Employee Creativity	0.000	0.057	0.006	0.497
Openness → Employee Creativity	0.213	0.052	4.079	0.000

**Table 6:** Discriminate validity.

	Agre	Cons	Ecre	Extra	Neur	Open
Agreeableness	*0.845*					
Conscientiousness	0.778	*0.855*				
Employee creativity	0.349	0.380	*0.752*			
Extraversion	0.014	0.028	0.038	*0.913*		
Neuroticism	0.712	0.663	0.316	0.036	*0.853*	
Openness	0.417	0.423	0.344	0.039	0.500	*0.837*

Italic values indicates square root of the AVE.

**Table 7:** Path coefficients.

	*β*	STDEV	*t* statistics	*p* values
BFPT → E_Creativity	0.429	0.066	6.455	0.000
Gender x BFPT → E_Creativity	−0.053	0.108	0.487	0.313

The findings of higher order construct (reflective-reflective) revealed that Big Five personality traits have a strong positive relationship with creativity (*β* = 0.429, *p* = 0.000), emphasizing their role in fostering innovative and original thinking. Gender is also explored as a moderating variable, suggesting that the strength and direction of these relationships may differ based on gender differences. The results indicated that gender does not have a significant moderating effect on relationship between Big Five personality traits and employee creativity (*β* = −0.053, *p* = 0.313).

## 5 Discussion

Researchers have long investigated the link between personality traits and creative behaviors through empirical studies, yet evidence from field studies is still limited (Khaleefa et al., 1996; Sung and Choi, 2009). Considering the importance of creativity for innovation and societal progress, the aim of this study was to identify some of its antecedents. To achieve this, the research focused on examining how the dimensions of the Big Five personality traits influence employee creativity. The results suggest that agreeableness, conscientiousness, and openness are significant indicators of creativity, as supported by Yao and Li (2021). They found that openness to experience, conscientiousness, and agreeableness were positively associated with creative behavior. In a similar vein, Karwowski et al. (2013) identified positive associations between openness to experience, extraversion, and conscientiousness with both creative self-efficacy and creative personal identity. Previous studies have primarily emphasized openness to experience as a critical factor in understanding creativity (Kaufman et al., 2016; Tan et al., 2019). Karwowski et al. (2013) noted that while openness to experience emerged as the most significant predictor among those examined, its moderate effect size warrants further consideration.

When comparing the findings to other research, this study found further evidence that conscientiousness is a personality attribute that is most closely related to creativity. The role of conscientiousness in creative behavior is particularly interesting, given that research suggests it is most reliable predictor of task performance (Barrick and Mount, 1991). However, Jirásek and Sudzina (2020) found a negative relationship between conscientiousness and creativity among students from Denmark. According to the results of Zare and Flinchbaugh (2019) study, openness and extraversion are more superior predictors than conscientiousness. Zare and Flinchbaugh (2019) found that agreeableness and neuroticism did not significantly predict creativity. Likewise, Jirásek and Sudzina (2020) reported that openness has the most significant impact on creativity. This research found that employee creativity is best predicted by conscientiousness, agreeableness and openness, whereas neuroticism and extraversion had no significant influence which is supported by Jirásek and Sudzina (2020) that showed extraversion weakly increase creativity. Hence, the impact of personality traits on employee creativity shows inconsistencies because individuals possess different personalities, and various combinations of these traits affect individual behavior differently.

The connection between openness and creative success has been consistently proven (Batey et al., 2010; Christensen et al., 2014). Openness to experience is a personality trait that describes individuals who are intellectually inquisitive and inclined to seek out new experiences and explore innovative ideas (Zhao and Seibert, 2006). It has been discovered that employees with open personalities are the most effective innovators in their roles. They seek out novel methods to complete tasks, explore creative solutions to work-related challenges, and look for new ideas to enhance work processes (Abou-Shouk et al., 2022). Numerous research has clearly established that openness to experience is the most important personality attribute (Furnham et al., 2013; Kaspi-Baruch, 2017; Silvia et al., 2014; Stock et al., 2016; Sung and Choi, 2009). The explanation is most likely that it directly represents creativity, as shown by its description (Jirásek and Sudzina, 2020). Previously, the incorporation of openness into the study of creativity has been recommended because of its positive impact (Tan et al., 2019). The connection between openness and creativity remains fairly consistent across different years and cultural settings (Christensen et al., 2014). Openness to experience was consistently and positively correlated with measures of creative potential, creative production, and self-report inventories that assessed multiple domains (Hornberg, 2022). Hence, the findings from this research offer further validation of Kaufman et al. (2016) claim that openness to experience is sole personality trait consistently correlated with creativity, based on the frequency of such findings. This implies that this relationship is not dependent on the presence or accessibility of specific theories in the literature (Christensen et al., 2014).

The results of the study indicated that extraversion did not provide statistically significant results with employee creativity which is line with previous studies (Kaspi-Baruch, 2017; Stock et al., 2016; Silvia et al., 2014). Similarly, Jirásek and Sudzina (2020) reported that extraversion’s two characteristics, assertiveness and activity, may not fully account for creativity. This may be due to the fact that, in certain cultures, creativity is more heavily influenced by conformity and institutional norms rather than by social assertiveness. In contrast, research by Pérez-Luño et al. (2023), Batey et al. (2010), Karwowski and Lebuda (2016) identified a positive link between extraversion and creativity. Christensen et al. (2014) indicate that extraversion is a potential predictor of creativity within divergent thinking (DT) frameworks. In comparison, agreeableness is a personality trait that has not consistently been connected to creativity, with most studies reporting insignificant results (Batey et al., 2010; Furnham et al., 2013). Nevertheless, this study’s results indicated a significant and positive correlation between agreeableness and creativity, a finding that is consistent with Kaufman et al. (2016).

As noted by Abou-Shouk et al. (2022), employees who exhibit extroversion and agreeableness are likely to enhance their organization’s performance. Puryear et al. (2017) meta-analysis found a positive correlation between extraversion and creativity. A significant relationship between conscientiousness and creativity was also observed. Further, conscientiousness pertains to an individual’s degree of organization, perseverance, effort, and drive toward goal achievement (Zhao and Seibert, 2006). The results of this study supported by Karwowski and Lebuda (2016) and Silvia et al. (2014) that reported statistically significant findings. Finally, this study did not find evidence to support a link between neuroticism and employee creativity. Similarly, Yao and Li (2021) suggested that neuroticism is not significantly related to creative behavior. In earlier studies, neuroticism was shown to be the attribute least associated with creativity (Pérez-Luño et al., 2023). Karwowski and Lebuda (2016) claimed that neuroticism have the strongest negative relations with creative self-efficacy (CSE) and creative personal identity (CPI). Our observation of no significant correlation might be due to the relatively low levels of neuroticism among the academic staff in our sample. Neuroticism does not greatly influence the creativity of academic staff, as academic creativity is more dependent on traits like openness, persistence, and intellectual engagement rather than emotional instability. The fear, anxiety, and excessive thinking linked to neuroticism can hinder creative risk-taking, making it less pertinent in academic contexts. This study suggests that future studies explore the relationship between neuroticism and creative behavior.

While research on the Big Five personality characteristics and employee creativity is extensive, studies that expressly include gender as a moderator are uncommon (Zare and Flinchbaugh, 2019). This study aims to explore the variations in creativity scores between males and females within a Sudanese sample. The findings of this study appear to contradict previous research that has reported gender differences in personality traits associated with creativity (Baer and Kaufman, 2008; Laouiti et al., 2022; Pérez-Luño et al., 2023). In this study, gender’s moderation effect on Big Five personality traits (BFPT) and employee creativity is not significant due to a small path coefficient (*β* = −0.053), a *t*-statistic (0.487) below the critical threshold (typically 1.96 for 95% confidence), and a *p*-value (0.313) greater than 0.05. The results of the moderation analysis showed that gender did not influence the relationship between personality traits and creativity. The lack of significant gender moderation indicates that gender does not significantly influence how personality traits affect creativity among Sudanese academics. The result is supported by Zare and Flinchbaugh (2019) and Kogan (1974) who reported no gender differences were found in terms of creativity. In other words, the predictive power of personality traits on creativity is consistent across genders.

In contrast to our findings, Karwowski et al. (2013) identified some intriguing differences. They discovered that among women, creative self-efficacy was positively associated with Extraversion and negatively with Agreeableness, whereas these associations were not found in men. Although the differences in perceived creative self-efficacy between genders are generally minor, they tend to favor men (Beghetto, 2006; Karwowski, 2011). Men not only view their creativity as being at a higher level but also tend to overrate it, whereas women often underestimate their creative self-efficacy (Karwowski, 2011). As highlighted by Karwowski et al. (2013), women achieve significant and exceptional creative accomplishments less frequently than men. This disparity may be partly due to their lack of belief in their own creative abilities. According to the findings of Pérez-Luño et al. (2023), women with higher levels of extraversion tend to exhibit greater creativity. The statistical analysis further indicated that high levels of openness to experience are beneficial for both men and women, with women utilizing these traits to a greater extent than men, as noted by Pérez-Luño et al. (2023). Khaleefa et al. (1996) discovered that men significantly surpassed women in terms of creativity. These varying results might be due to social and cultural influences. Another potential reason could be the disparity in educational opportunities traditionally available to each gender. Nonetheless, in present-day Sudan, education is equally accessible to both males and females at the same level.

By using a gender-based method to identify the impact of combination personality traits in the Sudanese environment, this study builds on earlier research on the factors that influence employee creativity. The subject reopens old debates about how society stereotypes gender relationships when it comes to creative issues (Baer and Kaufman, 2008; Pérez-Luño et al., 2023). The belief that men are the primary drivers of creativity has been prevalent in the past (Hmieleski and Sheppard, 2019), and remnants of this gender bias remain in contemporary society (Mensa and Grow, 2022). It has been argued that women are often at a disadvantage in creative fields, especially in developing nations like Sudan, because the label ‘creative genius’ is more commonly attributed to men than to women (Pérez-Luño et al., 2023). Nonetheless, these inconsistent results underscore the necessity for additional replication in upcoming studies.

## 6 Implications

### 6.1 Theoretical implications

The conceptual model developed in this study has some theoretical implications. Firstly, this research contributes to the existing literature on personality traits by investigating its impact on employee creativity while considering gender differences in Sudanese higher education. While most studies on personality and creativity have been conducted in Western contexts (Batey et al., 2010; Karwowski, 2011), this study broaden the scope by providing empirical insights from a developing country with distinct cultural and educational structures. Although earlier research has examined how individual factors affect creativity among students (Batey et al., 2010; Kaufman, 2006; Samašonok and Juškevičienė, 2022), there is a notable absence of studies focusing on academic staff, revealing a significant gap in the literature. Since faculty members are crucial in generating knowledge, fostering innovation, and developing curricula, it is vital to understand what influences their creativity to improve teaching methods, research productivity, and institutional innovation.

Secondly, this research advances the study of creativity and personality by integrating both lower-order and higher-order analyses. While earlier research has primarily focused on examining individual personality traits separately (lower-order analysis), this study’s inclusion of higher-order modeling offers a more complete understanding of how personality traits collectively impact creativity. By combining both levels of analysis, this research provides a more comprehensive theoretical framework and improves the precision of empirical results.

Finally, in accordance with Trait Activation Theory (Tett and Burnett, 2003) personality traits impact behavior in different ways depending on the work environment. This research adds to existing literature by exploring the role of gender as a moderating factor in the link between personality traits and creativity among Sudanese academics. The results revealed that gender does not play a significant moderating role in this relationship. This absence of significant gender moderation implies that both male and female academics cultivate creativity based on their professional duties rather than gender-specific personality differences. This finding challenges the conventional belief that creativity is influenced by gender. In a culturally rich society like Sudan, where Arab and African traditions converge, societal norms have historically shaped gender roles and expectations. Traditionally, men are often encouraged to assume leadership positions and engage in problem-solving activities that might foster creative expression. However, within the realm of higher education, where academic staff—regardless of gender—are provided with equal opportunities for research, teaching, and innovation, these traditional gender roles may become less pertinent.

### 6.2 Managerial implications

The results of this research provide important insights for universities, academic policymakers, and higher education administrators aiming to boost faculty creativity. Given that gender does not play a significant role in moderating the link between personality traits and creativity, institutions should prioritize creating an inclusive and supportive academic atmosphere that promotes creativity among faculty members, regardless of gender. This can be accomplished by ensuring equal access to research funding, innovation grants, and professional development opportunities that cultivate creative thinking and problem-solving abilities. Further, the findings could provide females with better strategies for managing their position in the labor market, while also demonstrating to employers and policymakers the significance of females holding creative positions. It underscores that women are just as capable of creativity as men, directly challenging the stereotypes that position men as the primary creators. By discrediting these biases, employers and policymakers can focus on more relevant factors when assessing an individual’s potential for creativity, regardless of gender.

Moreover, universities should establish specialized training programs that capitalize on personality-based strengths to enhance creative capabilities. For instance, faculty members who exhibit a high level of openness to experience can be motivated to participate in interdisciplinary research, whereas those with a strong sense of conscientiousness might thrive in structured innovation initiatives. Faculty members who score high in agreeableness are typically cooperative, supportive, and team-oriented, making them ideal candidates for collaborative research, mentorship roles, and community engagement projects. By acknowledging and harnessing a variety of personality traits, institutions can foster an academic environment that is both collaborative and innovation-focused.

## 7 Limitations

This study is not free from limitations. A significant drawback of this study is its reliance on cross-sectional data, as it lacks a longitudinal approach. Consequently, the research captures personality traits and creativity at a single moment, rather than observing their progression and changes over time. Future studies should explore longitudinal or experimental methodologies to gain deeper insights into the dynamic nature of personality traits, creativity, and their interplay within higher education environments. Another potential limitation of the instrument could be noted. Participants in this research used validated online tools in the form of a survey. The data collected from these surveys were self-reported, making independent verification challenging. Consequently, there might have been biases in participant responses, such as inaccuracies or a tendency to give socially desirable answers. In this study, the focus was limited to assessing how the Big Five personality traits directly influence employee creativity. Future research could expand by including other personality traits. Moreover, it would be worthwhile to explore if the Big Five traits have an indirect effect on creativity through intrinsic motivation.

## Data Availability

The original contributions presented in the study are included in the article/supplementary material, further inquiries can be directed to the corresponding author.

## References

[ref1] Abdullah I. Omar R. Panatik S. A. (2016). A literature review on personality, creativity and innovative behavior. Int. Rev. Manag. Mark. 6, 177–182.

[ref2] Abou-Shouk M. Zoair N. Aburumman A. Abdel-Jalil M. (2022). The effect of personality traits and knowledge-sharing on employees' innovative performance: a comparative study of Egypt and Jordan. Tour. Manag. Perspect. 44:101024. doi: 10.1016/j.tmp.2022.101024, PMID: 40104671

[ref3] Abra J. Valentine-French S. (1991). Gender differences in creative achievement: a survey of explanations. Genetic Soc. Gen. Psychol. Monogr. 117, 233–284, PMID: 1789886

[ref4] Afu M. (2020). Gender differences in teachers’ attitudes towards creative students in the Federal Capital Territory, Abuja-Nigeria. Int. J. Creat. Res. Thoughts 8, 740–746.

[ref5] Alblooshi M. (2018). Assessing factors that influence employees’ creativity in public-sector organisations. The case of Dubai government organisations. (Doctoral dissertation, University of Wollongong in Dubai).

[ref6] Alencar E. M. L. S. Fleith D. S. Pereira N. (2017). Creativity in higher education: challenges and facilitating factors. Temas em Psicologia 25, 553–561. doi: 10.9788/tp2017.2-09

[ref7] Al-Srour N. H. Al-Oweidi A. (2013). The level of creativity among management employees, academic staff and artistes and its relationship with gender, practical experience and age. Creative Educ. 4, 185–188. doi: 10.4236/ce.2013.43027

[ref8] Amabile T. M. (2017). In pursuit of everyday creativity. J. Creat. Behav. 51, 335–337. doi: 10.1002/jocb.200

[ref9] Amabile T. M. Barsade S. G. Mueller J. S. Staw B. M. (2005). Affect and creativity at work. Adm. Sci. Q. 50, 367–403. doi: 10.2189/asqu.2005.50.3.367

[ref10] Amin A. Basri S. Rehman M. Capretz L. F. Akbar R. Gilal A. R.. (2020). The impact of personality traits and knowledge collection behavior on programmer creativity. Inf. Softw. Technol. 128:106405. doi: 10.1016/j.infsof.2020.106405

[ref11] Baer J. Kaufman J. C. (2008). Gender differences in creativity. J. Creat. Behav. 42, 75–105. doi: 10.1002/j.2162-6057.2008.tb01289.x

[ref12] Bagozzi R. P. Yi Y. Phillips L. W. (1991). Assessing construct validity in organizational research. Adm. Sci. Q. 36, 421–458. doi: 10.2307/2393203

[ref13] Barańczuk U. (2019). The five factor model of personality and emotion regulation: a meta-analysis. Pers. Ind. Diff. 139, 217–227. doi: 10.1016/j.paid.2018.11.025

[ref14] Barrick M. R. Mount M. K. (1991). The big five personality dimensions and job performance: a meta-analysis. Pers. Psychol. 44, 1–26. doi: 10.1111/j.1744-6570.1991.tb00688.x

[ref15] Batey M. Chamorro-Premuzic T. Furnham A. (2010). Individual differences in ideational behavior: can the big five and psychometric intelligence predict creativity scores? Creat. Res. J. 22, 90–97. doi: 10.1080/10400410903579627

[ref16] Beghetto R. A. (2006). Creative self-efficacy: correlates in middle and secondary students. Creat. Res. J. 18, 447–457. doi: 10.1207/s15326934crj1804_4, PMID: 36589895

[ref18] Chiang Y.-H. Hsu C.-C. Shih H.-A. (2017). Extroversion personality, domain knowledge, and the creativity of new product development engineers. Creat. Res. J. 29, 387–396. doi: 10.1080/10400419.2017.1376501

[ref19] Christensen B. T. Drewsen L. K. Maaløe J. (2014). Implicit theories of the personality of the ideal creative employee. Psychol. Aesthet. Creat. Arts 8, 189–197. doi: 10.1037/a0036197

[ref20] Collins J. Cooke D. K. (2013). Creative role models, personality and performance. J. Manag. Dev. 32, 336–350. doi: 10.1108/02621711311326347

[ref21] Costa P. T. Jr. McCrae R. R. J. P. (1992). Four ways five factors are basic. Pers. Ind. Diff. 13, 653–665. doi: 10.1016/0191-8869(92)90236-I

[ref22] Dongell C. (2021). Creativity in a changing world: how personality, work environment, and flexibility affect employee creativity. (Doctoral dissertation, Arizona State University).

[ref23] Duan W. Tang X. Li Y. Cheng X. Zhang H. (2020). Perceived organizational support and employee creativity: the mediation role of calling. Creat. Res. J. 32, 403–411. doi: 10.1080/10400419.2020.1821563

[ref24] Farah Bakhiet S. Mohamed H. (2022). Gifted education in Sudan: reviews from a learning-resource perspective. Cogent Educ. 9:2034246. doi: 10.1080/2331186x.2022.2034246

[ref25] Fornell C. Larcker D. F. (1981). Evaluating structural equation models with unobservable variables and measurement error. J. Mark. Res. 18, 39–50. doi: 10.1177/002224378101800104

[ref26] Furnham A. Hughes D. J. Marshall E. (2013). Creativity, OCD, narcissism and the big five. Thinking Skills Creat. 10, 91–98. doi: 10.1016/j.tsc.2013.05.003

[ref27] Goldberg L. R. (1992). The development of markers for the big-five factor structure. Psychol. Assess. 4, 26–42. doi: 10.1037/1040-3590.4.1.26, PMID: 40095981

[ref28] Hair J. F. (2011). Multivariate data analysis: an overview. Int. Encycl. Stat. Sci., 904–907. doi: 10.1007/978-3-642-04898-2_395

[ref29] Hair J. Joe F. Sarstedt M. Matthews L. M. Ringle C. M. (2016). Identifying and treating unobserved heterogeneity with FIMIX-PLS: part I–method. Eur. Bus. Rev. 28, 63–76. doi: 10.1108/EBR-09-2015-0094

[ref30] Henseler J. Ringle C. M. Sarstedt M. (2016). Testing measurement invariance of composites using partial least squares. Int. Mark. Rev. 33, 405–431. doi: 10.1108/IMR-09-2014-0304

[ref31] Hibatallah S. Rahman S. A. (2020). Change and development of higher education in Sudan. Pesa Int. J. Soc. Stud. 6, 113–120. doi: 10.25272/j.2149-8385.2020.6.2.02

[ref32] Hmieleski K. M. Sheppard L. D. (2019). The Yin and Yang of entrepreneurship: gender differences in the importance of communal and agentic characteristics for entrepreneurs' subjective well-being and performance. J. Bus. Ventur. 34, 709–730. doi: 10.1016/j.jbusvent.2018.06.006

[ref33] Hong M. Dyakov D. G. Zheng J. (2020). Self-esteem and psychological capital: their mediation of the relationship between big five personality traits and creativity in college students. J. Psychol. Afr. 30, 119–124. doi: 10.1080/14330237.2020.1744286

[ref34] Hornberg J. R. (2022). Measures of creativity and the big five personality traits: an examination using relative importance analysis (Doctoral dissertation): University of Nebraska.

[ref35] Irmscher M. (2019). The interface function of thinking styles between personality and intelligence. World J. Educ. 9, 79–91. doi: 10.5430/wje.v9n1p79

[ref36] Jirásek M. Sudzina F. (2020). Big five personality traits and creativity. Qual. Innov. Pros. 24, 90–105. doi: 10.12776/qip.v24i3.1509

[ref37] Karwowski M. (2011). It doesn't hurt to ask… but sometimes it hurts to believe: polish students' creative self-efficacy and its predictors. Psychol. Aesthet. Creat. Arts 5, 154–164. doi: 10.1037/a0021427

[ref38] Karwowski M. Lebuda I. (2016). The big five, the huge two, and creative self-beliefs: a meta-analysis. Psychol. Aesthet. Creat. Arts 10, 214–232. doi: 10.1037/aca0000035

[ref39] Karwowski M. Lebuda I. Wisniewska E. Gralewski J. (2013). Big five personality traits as the predictors of creative self-efficacy and creative personal identity: does gender matter? J. Creat. Behav. 47, 215–232. doi: 10.1002/jocb.32

[ref40] Kaspi-Baruch O. (2017). Big five personality and creativity: the moderating effect of motivational goal orientation. J. Creat. Behav. 53, 325–338. doi: 10.1002/jocb.183, PMID: 40104209

[ref41] Kaufman J. C. (2006). Self-reported differences in creativity by ethnicity and gender. Appl. Cogn. Psychol. 20, 1065–1082. doi: 10.1002/acp.1255

[ref42] Kaufman J. C. Arrington K. F. Barnett P. J. Holinger M. Liu X. Xie L. (2022). Creativity is our gig: focusing on the positive and practical. Transl. Iss. Psychol. Sci. 8, 137–152. doi: 10.1037/tps0000298

[ref43] Kaufman J. C. Beghetto R. A. (2013). Do people recognize the four Cs? Examining layperson conceptions of creativity. Psychol. Aesthet. Creat. Arts 7, 229–236. doi: 10.1037/a0033295

[ref44] Kaufman S. B. Quilty L. C. Grazioplene R. G. Hirsh J. B. Gray J. R. Peterson J. B.. (2016). Openness to experience and intellect differentially predict creative achievement in the arts and sciences. J. Pers. 84, 248–258. doi: 10.1111/jopy.12156, PMID: 25487993 PMC4459939

[ref45] Kavirayani K. (2018). Historical perspectives on personality–the past and current concept: the search is not yet over. Arch. Med. Health Sci. 6, 180–186. doi: 10.4103/amhs.amhs_63_18

[ref46] Khaleefa O. H. Erdos G. Ashria I. H. (1996). Gender and creativity in an afro-Arab Islamic culture: the case of Sudan. J. Creat. Behav. 30, 52–60. doi: 10.1002/j.2162-6057.1996.tb00757.x, PMID: 40109066

[ref47] Kim Nam N. Thi Hang Nga N. (2024). Influence of personality traits on creativity and innovative work behavior of employees. Probl. Perspect. Manag. 22, 389–398. doi: 10.21511/ppm.22(2).2024.30

[ref48] Kogan N. (1974). Creativity and sex differences. The. J. Creat. Behav. 8, 1–14. doi: 10.1002/j.2162-6057.1974.tb01103.x

[ref49] Kruyen P. M. van Genugten M. (2017). Creativity in local government: definition and determinants. Public Adm. 95, 825–841. doi: 10.1111/padm.12332, PMID: 40103450

[ref50] Laouiti R. Haddoud M. Y. Nakara W. A. Onjewu A.-K. E. (2022). A gender-based approach to the influence of personality traits on entrepreneurial intention. J. Bus. Res. 142, 819–829. doi: 10.1016/j.jbusres.2022.01.018

[ref51] Liao H. Liu D. Loi R. (2010). Looking at both sides of the social exchange coin: a social cognitive perspective on the joint effects of relationship quality and differentiation on creativity. Acad. Manag. J. 53, 1090–1109. doi: 10.5465/amj.2010.54533207

[ref52] Malok M. N. (2012). The relationship between leadership style and motivation among faculty members in two public universities in the republic of South Sudan: University of the Incarnate Word.

[ref53] Manteli M. Galanakis M. (2022). The new foundation of organizational psychology. Trait activation theory in the workplace: literature review. J. Psychol. Res. 12, 939–945. doi: 10.17265/2159-5542/2022.12.004

[ref54] Mark R. (1996). Research made simple: a handbook for social workers: Sage.

[ref55] McCrae R. R. Costa P. T. Jr. (1989). More reasons to adopt the five-factor model, vol. 44, 451–452 doi: 10.1037/0003-066X.44.2.451

[ref56] Mensa M. Grow J. M. (2022). “Now I can see”: creative women fight against machismo in Chilean advertising. Gender Manag. Int. J. 37, 405–422. doi: 10.1108/GM-04-2021-0098

[ref57] Munir R. Beh L.-S. (2016). Do personality traits matter in fostering innovative work behavior. Soc. Sci. 11, 4393–4398.

[ref58] Mutlu M. D. (2017). The role of personality composition on team creativity and innovation. (Doctoral dissertation, University of Sheffield).

[ref9001] Nguyen K. N. Nga N. T. H. (2024). Influence of personality traits on creativity and innovative work behavior of employees. Problems and Perspectives in Management 22:405.

[ref59] Osman Z. Senathirajah A. Rasheedul Haque R. Khalil M. (2024). A structural equation modelling approach on determinants of working adults' choice to further study in Malaysian online distance learning higher education institution. Educ. Admin. Theory Pract. 30, 20–31.

[ref60] Pérez-Luño A. Aguilar-Caro R. Muñoz-Doyague M. F. (2023). The influence of personality and team-member exchange on creativity: a gendered approach. Gender Manag. Int. J. 39, 145–164. doi: 10.1108/gm-01-2022-0023, PMID: 35579975

[ref61] Pett M. A. Lackey N. R. Sullivan J. J. (2003). Making sense of factor analysis: the use of factor analysis for instrument development in health care research: Sage.

[ref62] Pressman M. (2016). Chapter 3: Correlational research design and analysis. GCU doctoral research: quantitative qualitative research concepts.

[ref63] Puryear J. S. Kettler T. Rinn A. N. (2017). Relationships of personality to differential conceptions of creativity: a systematic review. Psychol. Aesthet. Creat. Arts 11, 59–68. doi: 10.1037/aca0000079

[ref64] Puryear J. S. Kettler T. Rinn A. N. (2019). Relating personality and creativity: considering what and how we measure. J. Creat. Behav. 53, 232–245. doi: 10.1002/jocb.174

[ref65] Qahl M. S. A. Hawryszkiewycz Igor Binsawad Muhammad Rehman J. (2019). Factors affecting the Saudi Arabian higher education creative environment. In 30th Australasian conference on information systems.

[ref66] Rothmann S. Coetzer E. P. (2003). The big five personality dimensions and job performance. SA J. Ind. Psychol. 29, 68–74. doi: 10.4102/sajip.v29i1.88, PMID: 40062343

[ref67] Samašonok K. Juškevičienė A. (2022). Perceived creativity and the big five personality traits of specialists trained for profession: trends in their interaction. Entrepr. Sustain. Iss. 9, 265–283. doi: 10.9770/jesi.2022.9.3(16)

[ref68] Seo J. (2021). How do organizations address the tension between codes of ethics and employee creativity? The role of ethics training: University of Minnesota.

[ref69] Silver J. R. Roche S. P. Bilach T. J. Bontrager Ryon S. (2017). Traditional police culture, use of force, and procedural justice: investigating individual, organizational, and contextual factors. Justice Q. 34, 1272–1309. doi: 10.1080/07418825.2017.1381756

[ref70] Silvia P. J. Beaty R. E. Nusbaum E. C. Eddington K. M. Levin-Aspenson H. Kwapil T. R. (2014). Everyday creativity in daily life: an experience-sampling study of “little c” creativity. Psychol. Aesthet. Creat. Arts 8, 183–188. doi: 10.1037/a0035722

[ref71] Stevenson C. Baas M. van der Maas H. (2021). A minimal theory of creative ability. J. Intelligence 9:9. doi: 10.3390/jintelligence9010009, PMID: 33669216 PMC8006236

[ref72] Stock R. M. von Hippel E. Gillert N. L. (2016). Impacts of personality traits on consumer innovation success. Res. Policy 45, 757–769. doi: 10.1016/j.respol.2015.12.002

[ref73] Stritmatter A. N. (2023). Personality traits, creativity, and innovation among police officers: a quantitative correlational study. (Doctoral dissertation): Grand Canyon University.

[ref74] Suifan T. S. Abdallah A. B. Al Janini M. (2018). The impact of transformational leadership on employees’ creativity: the mediating role of perceived organizational support. Manag. Res. Rev. 41, 113–132. doi: 10.1108/mrr-02-2017-0032

[ref75] Sung S. Y. Choi J. N. (2009). Do big five personality factors affect individual creativity? The moderating role of extrinsic motivation. Soc. Behav. Pers. Int. J. 37, 941–956. doi: 10.2224/sbp.2009.37.7.941

[ref76] Tan C. S. Lau X. S. Kung Y. T. Kailsan R. A. L. (2019). Openness to experience enhances creativity: the mediating role of intrinsic motivation and the creative process engagement. J. Creat. Behav. 53, 109–119. doi: 10.1002/jocb.170

[ref77] Tett R. P. Burnett D. D. (2003). A personality trait-based interactionist model of job performance. J. Appl. Psychol. 88, 500–517. doi: 10.1037/0021-9010.88.3.500, PMID: 12814298

[ref78] Tsai C.-F. Chang C.-P. Chen T.-L. Hsu M.-L. (2024). Exploring the influence of personality traits, self-efficacy, and creativity on employability for hospitality and tourism college students. Sustain. For. 16:1490. doi: 10.3390/su16041490

[ref79] Tussey K. N. (2023). Relationships between big five personality traits and three dimensions of employee engagement. (Doctoral dissertation): Franklin University.

[ref81] Wasko M. M. Faraj S. (2005). Why should I share? Examining social capital and knowledge contribution in electronic networks of practice. MIS Q. 29, 35–57. doi: 10.2307/25148667

[ref82] Yao X. Li R. (2021). Big five personality traits as predictors of employee creativity in probation and formal employment periods. Personal. Individ. Differ. 182:109914. doi: 10.1016/j.paid.2020.109914

[ref83] Yesil S. Sozbilir F. (2013). An empirical investigation into the impact of personality on individual innovation behaviour in the workplace. Proc. Soc. Behav. Sci. 81, 540–551. doi: 10.1016/j.sbspro.2013.06.474, PMID: 40104671

[ref84] Zare M. Flinchbaugh C. (2019). Voice, creativity, and big five personality traits: a meta-analysis. Hum. Perform. 32, 30–51. doi: 10.1080/08959285.2018.1550782

[ref85] Zhao H. Seibert S. E. (2006). The big five personality dimensions and entrepreneurial status: a meta-analytical review. J. Appl. Psychol. 91, 259–271. doi: 10.1037/0021-9010.91.2.259, PMID: 16551182

